# A prospective cohort study of cigarette smoking and the risk of endometrial cancer

**DOI:** 10.1038/sj.bjc.6600278

**Published:** 2002-05-06

**Authors:** P D Terry, A B Miller, T E Rohan

**Affiliations:** Department of Epidemiology and Social Medicine, Albert Einstein College of Medicine, Bronx, New York, New York, USA; Department of Public Health Sciences, University of Toronto, Toronto, Canada; Division of Clinical Epidemiology, Deutsches Krebsforschungszentrum, Heidelberg, Germany

**Keywords:** smoking, endometrial neoplasms, oestrogens, menopause, cohort studies

## Abstract

Case–control studies have shown inverse associations between cigarette smoking and endometrial cancer risk. However, two small prospective cohort studies have not clearly supported an association. Moreover, quantitative measures of smoking have been examined infrequently. Our aim was to study the association between smoking and endometrial cancer risk in a large prospective cohort. We used proportional hazards models to estimate hazard ratios relating cigarette smoking to endometrial cancer risk among 70 591 women aged 40–59 years at recruitment into a randomised controlled trial of mammography screening for breast cancer. During an average of 10.6 years of follow-up (751 833 person–years), a total of 403 women were diagnosed with incident endometrial cancer. We found that a reduced endometrial cancer risk was evident only among women who currently smoked 20 cigarettes per day or more (hazard ratio=0.62, 95% CI=0.42–0.92, *P* for trend=0.03). There was some suggestion of an inverse association with smoking duration, but this was less clear. The association did not vary with menopausal status, relative body weight, or the use of hormone replacement therapy, but it appeared to be stronger among parous than nulliparous women. The underlying biological mechanisms of this association remain unclear.

*British Journal of Cancer* (2002) **86**, 1430–1435. DOI: 10.1038/sj/bjc/6600278 www.bjcancer.com

© 2002 Cancer Research UK

## 

Endometrial cancer is the most commonly diagnosed cancer of the female genital tract in developed countries (Parker *et al*, 1997). Cigarette smoking has been associated inversely with endometrial cancer risk, but only in case–control studies. Although the mechanism through which smoking may reduce risk is unknown, it has been postulated that it might occur through a reduction in the gastrointestinal absorption or distribution of oestrogen, or through increased hepatic metabolism of oestrogen, oestrogen being one of the few known risk factors for this malignancy (Baron, 1984).

Although the number of studies that address this issue is growing, many aspects of the relationship between cigarette smoking and endometrial cancer risk remain under-explored. For example, most previous studies have not directly examined smoking intensity or smoking duration in relation to endometrial cancer risk. The examination of these smoking measures is important, since potentially deleterious effects of the many carcinogens contained in tobacco smoke (IARC, 1986; Hoffmann and Hoffmann, 1997) may be observed only with smoking of long duration, such as with cancers of the colorectum (Giovannucci, 2001), or with smoking of high intensity. Furthermore, if a dose-response association with cigarette smoking can be demonstrated with increasing levels of a particular smoking measure, the role of smoking in endometrial cancer etiology may be established more firmly.

Prospective cohort studies of smoking and endometrial cancer risk, in which the problems of selection and recall bias are minimised, have been scarce. Indeed, the only two such studies (Engeland *et al*, 1996; Terry *et al*, 1999) were relatively small and uninformative. We have therefore examined the relation between cigarette smoking and risk of endometrial cancer in a cohort of women with up to four decades of smoking duration at recruitment, who were subsequently followed for an average of 10.6 years.

## SUBJECTS AND METHODS

### Study population

The investigation was conducted using data from the Canadian National Breast Screening Study (NBSS). The NBSS is a multi-centre randomised controlled trial of mammography screening for breast cancer in 89 835 women aged 40–59 years at recruitment (Miller *et al*, 1992). Participants were recruited between 1980 and 1985 by various means, including personal invitation by letter, group mailings to employees of large institutions and to members of professional associations, advertisements in newspapers, and public service announcements on radio and television.

### Questionnaires

On enrolment in the NBSS, all participants completed a self-administered questionnaire that sought data on demographic characteristics, lifestyle (including cigarette smoking), menstrual and reproductive history, use of oral contraceptives and replacement oestrogens. Regarding smoking history, participants were first asked whether or not they had ever smoked. Women who had smoked were then asked how many cigarettes they smoked per day, for how many years they had smoked, and the year they had ceased smoking (former smokers only). Starting in 1982, a questionnaire regarding diet and physical activity was distributed to all new attendees at all screening centres, and to women returning to the screening centres for re-screening (Jain *et al*, 1982). By the time that the dietary questionnaire was introduced, some women had already been enrolled in the study and were not seen again at the screening centres. A total of 56 837 women returned completed dietary questionnaires. Therefore, to assess the possibility of residual confounding among women with missing information on alcohol consumption and physical activity, analyses were performed both on the entire cohort and among women for whom information on alcohol and physical activity was available (see below).

### Cohort and case definition and ascertainment of outcome

Women were eligible for inclusion in the cohort if, at recruitment, they had neither a history of endometrial cancer nor a history of hysterectomy. On this basis, 19 158 women (19 049 with history of hysterectomy, 109 with a history of endometrial cancer) were excluded, as were 86 women for whom no smoking information was available, and follow-up was based on the cohort of 70 591 women with intact uteri and with no history of endometrial cancer. Four hundred and three women with incident endometrial cancer were identified during the follow-up period. Cases of incident endometrial cancer and deaths were ascertained respectively through computerised record linkage to the Canadian Cancer Database and the National Mortality Database, both of which are maintained by Statistics Canada. There is good evidence from the NBSS and from other sources that the use of record linkage to ascertain incident cancer cases and deaths in Canada is both accurate and complete (Robles *et al*, 1988; Shannon *et al*, 1989).

### Statistical analysis

Follow-up of the cohort was continued until the date of diagnosis of endometrial cancer, the date of death, or the end of the follow-up period (December 31, 1993), whichever was the earliest. Cox proportional hazards models were used to estimate hazard ratios (RR) and 95% confidence intervals (CI) for the association between smoking and endometrial cancer risk. Age at smoking commencement was calculated for each smoker by subtracting her total years of smoking (and the time since quitting for ex-smokers) from her age at recruitment. Multivariate models included age in 5-year age groups, Quetelet's index (quartiles), education level (less than high school, high school, and university), vigorous physical activity (hours per day in tertiles, and ‘missing’), hormone replacement therapy (never + four levels of duration), oral contraceptive use (never + four levels of duration), menopausal status (pre, peri, post at recruitment), parity (nulliparous, and tertiles), and alcohol consumption (tertiles, and ‘missing’). Alcohol consumption and physical activity were categorised by tertiles or as ‘missing,’ where the latter group was comprised of women who did not complete the questionnaire regarding physical activity and diet. The lowest tertile of alcohol consumption was comprised of non-drinkers. To assess the possibility of residual confounding among women with missing information on alcohol consumption and physical activity, we conducted additional analyses limited to the 44 525 women (including 257 cases) who completed questionnaires regarding diet and physical activity and who did not have a history of hysterectomy. For tests of trend in risk across successive levels of categorical variables, median values of each category were fitted into the risk models as successive integers (Rothman and Greenland, 1998).

Analyses were conducted overall and within strata defined by menopausal status, obesity, and exogenous hormone use. Women who, at recruitment, reported having had regular menstrual periods within the past 12 months were considered to be premenopausal, while those in whom menstrual periods had ceased at least 12 months before baseline assessment (or before recruitment) were considered to be postmenopausal, as were women who had previously undergone oophorectomy (with or without a history of hysterectomy). For the purposes of stratified analyses with respect to obesity, women were categorised according to criteria for obesity established by the World Health Organisation, (WHO, 1997) namely ‘pre-obese’ (BMI 25.0−<30 kg m^−2^) and ‘obese’ (BMI ⩾30 kg m^−2^); those with a BMI <25.0 kg m^−2^ were classified as ‘non-obese.’

## RESULTS

On average, participants were followed for 10.6 years, yielding a total of 751 833 person-years of follow-up for the cohort. The average age at diagnosis of endometrial cancer was 57.4 years. Current smokers had the lowest median BMI, were the least likely to have completed secondary education, had the greatest percentage of hormone replacement therapy, were most likely to be postmenopausal, and along with former smokers had a higher percentage of oral contraceptive use and alcohol consumption than never smokers (Table 1

**Table 1:**
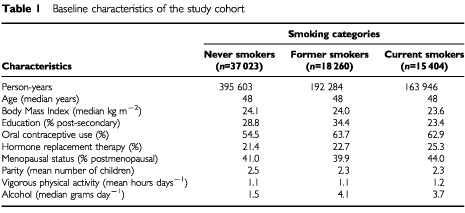
Baseline characteristics of the study cohort

). There were no clear differences by smoking status with respect to age, parity, or vigorous physical activity.

Current smokers had a 23% reduced risk of endometrial cancer after adjusting for the effect of age only, which was moderately attenuated by the addition of Quetelet's Index (kg m^−2^) to the model (Table 2

**Table 2:**
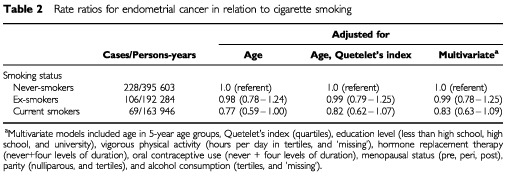
Rate ratios for endometrial cancer in relation to cigarette smoking

). The addition of other covariates to the model did not alter the rate ratios appreciably. No altered risk was observed among ex-smokers.

Multivariate-adjusted rate ratios for various measures of cigarette smoking are shown in Table 3

**Table 3:**
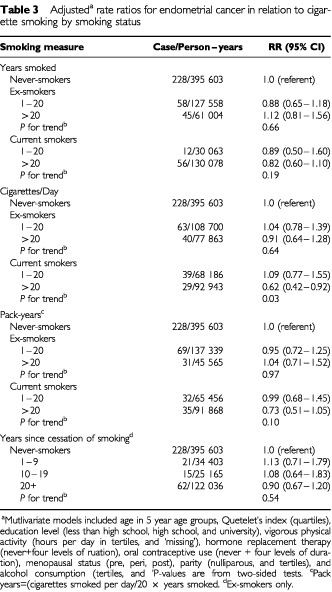
Adjusted^a^ rate ratios for endometrial cancer in relation to cigarette smoking by smoking status

for former and current smokers separately. The absence of an association among former smokers did not vary according to the total years or pack-years smoked, the number of cigarettes smoked per day, or with years since smoking ceased. Among current smokers, however, a reduced endometrial cancer risk was evident only among women who smoked 20 cigarettes per day or more; there was some suggestion of an inverse association with smoking duration, but this was less clear. In further analyses amongst current smokers, when smoking duration (1–19 and 20+ years, respectively) was examined over strata of smoking intensity (1–19 and 20+ cigarettes per day, respectively), a statistically significant 40% reduction in risk was observed only among women who were both long-term and heavy smokers RR=0.63, 95%CI=0.42–0.96). In current smokers, pack-years of consumption, the product of smoking duration and intensity, was inversely associated with endometrial cancer risk with a magnitude that was intermediate between those observed for the two latter measures (Table 3). There was a statistically non-significant 30% reduction in risk with commencement of smoking at age 15 or earlier (data not shown).

Among current smokers, the association between smoking intensity and endometrial cancer risk did not vary appreciably according to menopausal status, relative body weight, history of oral contraceptive use, or hormone replacement therapy (Table 4

**Table 4:**
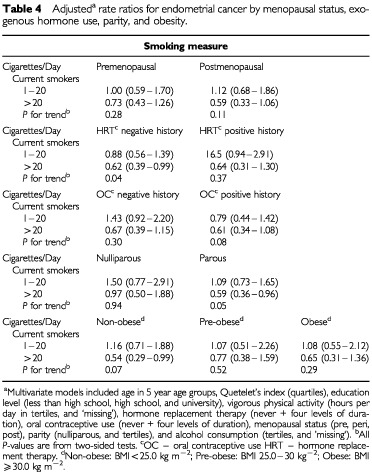
Adjusted^a^ rate ratios for endometrial cancer by menopausal status, exogenous hormone use, parity, and obesity

). Similarly, the association between smoking duration and risk did not vary over strata of these variables (data not shown). However, while there was an inverse association between smoking intensity and endometrial risk among parous current smokers, there was no association among nulliparous women, either with smoking intensity or duration, although the number of cases among nulliparous women was low (*n*=74) and the confidence intervals were wide. On formal testing, an interaction between smoking and parity in relation to endometrial cancer risk was not detected. The association between smoking and endometrial cancer risk did not vary according to levels of physical activity or alcohol consumption (data not shown). Among ex-smokers, there was no clear association with any of the smoking variables in any of the strata of the factors that were examined.

The results described above were similar to those obtained when the analyses were performed among the 44 525 women for whom alcohol consumption and physical activity data were available (data not shown); these women were similar to the remainder of the cohort with respect to the variables included in the multivariate models. The results were also largely unaltered after excluding cases that occurred within the first year of follow-up, thereby reducing the likelihood that changes in smoking habits due to pre-clinical undiagnosed endometrial cancer might have influenced our results.

## DISCUSSION

In our large prospective cohort study, we found that current smokers, but not former smokers, had a reduced risk of endometrial cancer. The inverse association among current smokers appeared to be confined to women who smoked one packet of cigarettes per day or more, a group which had also mainly smoked for 20 years or longer. The approximately 40% reduced risk among these women compared with never smokers did not vary appreciably according to strata defined by menopausal status, relative body weight, or exogenous hormone use, but the association appeared to be stronger among parous than nulliparous women. Our results suggest that a reduced relative body weight among current smokers, compared to never smokers and former smokers, explains some, but not all, of the inverse association between smoking and endometrial cancer risk. Although most studies have not specifically examined the contribution of body mass index (BMI) to the association between smoking and endometrial cancer risk, a recent case–control study (Weiderpass and Baron, 2001) found that adjustment for BMI attenuated the relative risk estimates for current smoking only slightly.

Among the strengths of our study was the large sample size of our cohort of women and the relatively long-term follow-up. The completeness of follow-up of the cohort (Robles *et al*, 1988; Shannon *et al*, 1989) reduces the likelihood that our results reflect bias due to differential follow-up of long-term smokers compared with non-smokers. However, we did not have information on hysterectomy occurrence during follow-up. Given the 20–130% higher risk of hysterectomy among smokers compared with non-smokers observed in a recent study (Harlow and Barbieri, 1999), a greater proportion of smokers than non-smokers would have continued to be followed (and to have contributed follow-up time) after they were no longer at risk of endometrial cancer, which would erroneously have lowered the rate of disease among smokers and biased results towards the finding of an inverse association. However, the estimated rate of hysterectomy in Canada at the time of follow-up was 62 per 10 000 person-years (Wijma *et al*, 1984), which, if unaccounted for, would have resulted in a relative inflation of person-time for smokers of approximately 5%. Correction for this in our data, using the estimate of 130% higher risk of hysterectomy among the heaviest smokers (Harlow and Barbieri, 1999), resulted in a change in the rate ratio for smoking 20 cigarettes per day or more from 0.62 to 0.65, a negligible difference. Finally, although we did adjust our estimates for a wide range of potentially confounding variables, we cannot exclude the possibility of residual confounding by other factors.

Factors that influence circulating levels of oestrogen are among the few established risk factors for endometrial cancer (Akhmedkhanov *et al*, 2001). These factors include a high relative body weight, low parity, and oestrogen replacement therapy. Oestrogen is thought to increase the risk by increasing the mitotic activity of endometrial cells, increasing the number of DNA replication errors, and by inducing somatic mutations resulting in the malignant phenotype (Akhmedkhanov *et al*, 2001). Hence, factors associated with reduced circulating oestrogen levels may consequently reduce the risk of this malignancy (Baron, 1984; Baron *et al*, 1990). In this regard, it has been hypothesised that smoking might be inversely related to levels of circulating oestrogen (Jensen *et al*, 1985; Baron *et al*, 1990; Weiderpass and Baron, 2001). Supporting this hypothesis are studies that have shown that smoking is associated with increased risk of osteoporosis (Jensen *et al*, 1985; Jensen and Christiansen, 1988) and may attenuate the reduction in serum total and low-density lipoprotein cholesterol associated with exogenous hormone use (HRT) (Jensen and Christiansen, 1988). However, plasma levels of oestradiol and oestrone have not been significantly associated with smoking either in pre- or postmenopausal women in several studies (Khaw *et al*, 1988; Longcope and Johnston, 1988; Baron *et al*, 1990; Cassidenti *et al*, 1992), although reduced levels of oestrone and oestradiol in current smokers compared with ex-smokers or never smokers have been noted (Austin *et al*, 1993), especially among women using HRT (Jensen *et al*, 1985; Cassidenti *et al*, 1990). Paradoxically, these studies have also found higher circulating levels of androstenedione among current than former smokers (Cassidenti *et al*, 1990; Austin *et al*, 1993), especially among women who were postmenopausal and/or obese.

Cigarette smoking has been associated inversely with endometrial cancer risk in both population-based (Tyler *et al*, 1985; Franks *et al*, 1987; Lawrence *et al*, 1987, 1989; Elliott *et al*, 1990; Rubin *et al*, 1990; Brinton *et al*, 1993; Newcomer *et al*, 2001; Weiderpass and Baron, 2001) and hospital-based (Williams and Horm, 1977; Baron, 1984; Lesko *et al*, 1985; Levi *et al*, 1987; Stockwell and Lyman, 1987; Koumantaki *et al*, 1989; Austin *et al*, 1993) case–control studies, although in several studies of both types the observed associations were weak and statistically non-significant. Of the two previous prospective studies of endometrial cancer incidence, one found a small, statistically non-significant increased risk with ‘current’ smoking (Engeland *et al*, 1996), and the results of the other (Terry *et al*, 1999) were also equivocal; both, however, were small with 36 and 12 cases respectively among current smokers.

In the previous examinations of risk in relation to smoking intensity (Lesko *et al*, 1985; Lawrence *et al*, 1987, 1989; Levi *et al*, 1987; Stockwell and Lyman, 1987; Brinton *et al*, 1993; Terry *et al*, 1999; Weiderpass and Baron, 2001), the highest category was generally at least 15 cigarettes per day or more and generally showed a 25–60% decreased risk among ‘current’ smokers but not among ex-smokers. In addition to smoking intensity, two studies (Brinton *et al*, 1993; Weiderpass and Baron, 2001) also examined risk in relation to smoking duration of up to 40 years or more. In contrast with our findings, smoking duration was more strongly associated with endometrial cancer risk than smoking intensity in those studies. However, the fact that the various smoking measures are often correlated with each other (as in our data) complicates the differentiation of their independent effects. In general, smokers of high intensity also tend to be smokers of long duration, tending to have commenced smoking at an early age. A non-significant reduced risk has been reported with early age at start of smoking (Brinton *et al*, 1993) as we also found; this aspect was not examined in other studies.

Several studies have shown a reduced endometrial cancer risk with current smoking that is stronger among, or limited to, postmenopausal women (Smith *et al*, 1984; Lesko *et al*, 1985; Brinton *et al*, 1993; Weiderpass and Baron, 2001), women using HRT (Weiss *et al*, 1980; Franks *et al*, 1987; Levi *et al*, 1987), and those who are obese (Lawrence *et al*, 1987; Brinton *et al*, 1993). However, as with our study, other studies have failed to demonstrate significant differences according to menopausal status (Lawrence *et al*, 1987; Levi *et al*, 1987), obesity (Levi *et al*, 1987), or HRT use (Lawrence *et al*, 1987; Brinton *et al*, 1993; Weiderpass and Baron, 2001), and at least three studies (Lawrence *et al*, 1987; Brinton *et al*, 1993; Weiderpass and Baron, 2001) found stronger inverse associations among non-users of HRT. Thus, whether factors related to circulating oestrogen levels modify the association between smoking and endometrial cancer risk remains unclear. It is interesting that we found smoking associated with risk only among parous women, although the number of cases among nulliparous women was relatively small. As in our study, previous studies have found a lower risk of endometrial cancer among parous than nulliparous women (Terry *et al*, 1999; Weiderpass and Baron, 2001), which may be due to significantly lower circulating oestrogen levels in the former (Bernstein *et al*, 1985).

In summary, we found that current smoking was associated with a reduced risk of endometrial cancer as in most previous case–control studies; risk was observed primarily among women who had smoked at least one packet of cigarettes per day for a relatively long time, but the association was not observed among former smokers. The mechanisms underlying this association remain unclear.
